# Supplementary material for: An interpretable machine learning algorithm enables dynamic 48-hour mortality prediction during an ICU stay
Source: Commun Med (Lond). 2025 Oct 15;5:426. doi: 10.1038/s43856-025-01192-z (PMC12528449; doi:10.1038/s43856-025-01192-z)
Supplement: Supplementary file 2 — Supplemantary Information [file 43856_2025_1192_MOESM2_ESM.docx]

**SUPPLEMENTARY INFORMATION**

Supplement to:

**An interpretable machine learning algorithm enables dynamic 48-hour mortality prediction during an ICU stay**

Simone Britsch, Markward Britsch et al.

**Table of contents**

Contents

[Supplemental Table 1: 2](#_Toc204798692)

[Supplemental Table 2 3](#_Toc204798693)

[Supplemental Table 3 4](#_Toc204798694)

[Supplemental Table 4 11](#_Toc204798695)

[Supplemental Table 5 13](#_Toc204798696)

[Supplemental Table 6 14](#_Toc204798697)

[Supplemental Table 7 15](#_Toc204798698)

[Supplemental Table 8 16](#_Toc204798699)

[Supplemental Table 9: 17](#_Toc204798700)

[Supplemental Table 10: 18](#_Toc204798701)

[Supplemental Table 11: 19](#_Toc204798702)

[Supplemental Table 12: 21](#_Toc204798703)

[Supplemental Figure 1 22](#_Toc204798704)

[Supplemental Figure 2: 23](#_Toc204798705)

[Supplemental Figure 3: 24](#_Toc204798706)

[Supplemental Figure 4: 25](#_Toc204798707)

[Supplemental Figure 5: 26](#_Toc204798708)

[Supplemental Figure 6: 27](#_Toc204798709)

Supplemental Table 1: *Categorization of patient stays into disease groups based on ICD-10 codes^1^.*

| **Diagnosis group** | **ICD-10 codes** |
| --- | --- |
| Diseases of the digestive system | K00-K93 |
| Diseases of the nervous system | G00-G99 |
| Injury, poisoning and certain other consequences of external causes | S00-S99, T00-T98 |
| Certain infectious and parasitic diseases | A00-A99, B00-B99 |
| Diseases of the circulatory system | I00-I99 |
| Neoplasms | C00-C99, D00-D48 |
| Diseases of the respiratory system | J00-J99 |
| Diseases of the genitourinary system | N00-N99 |
| Other | All other ICD-10 codes |

^1^According to ICD-10 code, International Classification of Diseases codes, 10th revision, German modification.

Supplemental Table 2: *Overview of variables, selected measurements ranges and count of excluded measurements.*

|  | **Measurement range** | **Excluded measurements, n (%)** |
| --- | --- | --- |
| **Demographics** |  |  |
| Age (years) | 18-130 | 0 (0.00) |
| Sex | W / M | 0 (0.00) |
| BMI (kg/m^2^) | 5-80 | 0 (0.00) |
| **Vital signs** |  |  |
| MAP (mmHg) | 10-250 | 2,868 (0.05) |
| Heart rate (/min) | 10-300 | 1,720 (0.03) |
| O_2_ saturation (%) | 10-100 | 7,984 (0.22) |
| Respiratory rate (/min) | 0-60 | 926 (0.10) |
| Body temperature (°C) | 25-45 | 4,420 (0.12) |
| Glasgow coma scale | 3-15 | 0 (0.00) |
| RASS | (-5)-4 | 0 (0.00) |
| Daily fluid balance (ml) | (-30,000)-30,000 | 6,890 (0.24) |
| **Labaratory parameters** |  |  |
| Lactate, arterial/venous (mg/dl) | 0-25 | 654 (0.05) |
| pH, arterial/venous | 10-500 | 330 (0.03) |
| Hemoglobin (g/dl) | 1-25 | 0 (0.00) |
| Thrombocytes (10^9^/l) | 0 | 0 (0.00) |
| Leukocytes (10^9^/l) | 0-150,000 | 0 (0.00) |
| C-reactive protein (mg/l) | 0-2,000 | 0 (0.00) |
| Creatinine (mg/dl) | 0-20 | 2 (0.00) |
| Blood urea nitrogen (mg/dl) | 0-1,500 | 0 (0.00) |
| Bilirubin (mg/dl) | 0-30 | 0 (0.00) |
| **Ventilation parameters** |  |  |
| PaO_2_/F_i_O_2_ (mmHg) | 10-600 | 2,952 (0.39) |
| PEEP (mb) | 0-40 | 40 (0.00) |
| Tidal volume (ml) | 0-2,000 | 4,766 (0.15) |
| **ICU scores** |  |  |
| SAPS II | 0-200 | 0 (0.00) |
| TISS-10 | 0-100 | 0 (0.00) |
| **Catecholamine dose** |  |  |
| Epinephrine (mg/24h) | no range | 0 (0.00) |
| Norepinephrine (mg/24h) | no range | 0 (0.00) |
| Dobutamine (mg/24h) | no range | 0 (0.00) |

BMI, body mass index. MAP, mean arterial pressure. PEEP, positive end-expiratory pressure. RASS, Richmond Agitation-Sedation Scale. SAPS II, Simplified Acute Physiology Score II. TISS-10, Therapeutic Intervention Scoring System.

Supplemental Table 3**:** *Overview of features included in the machine learning algorithms.*

| **Feature** | **Median/ number both** | **Median/ number test set** | **Median/ number training set** | **IQR/ potion both** | **IQR/ potion test set** | **IQR/ potion training set** | **Measured variable** | **Invalid values both** | **Invalid values test set** | **Invalid values training set** | **Invalid values both %** | **Invalid values test set %** | **Invalid values training set %** |
| --- | --- | --- | --- | --- | --- | --- | --- | --- | --- | --- | --- | --- | --- |
| Admission source cardiac catheterization lab | 758 | 382 | 376 | 1.24 % | 1.27 % | 1.21 % | Admission source | 965 | 457 | 508 | 0.0157672 | 0.0151470 | 0.0163702 |
| Admission source direct to ICU | 1936 | 976 | 960 | 3.16 % | 3.23 % | 3.09 % | Admission source | 965 | 457 | 508 | 0.0157672 | 0.0151470 | 0.0163702 |
| Admission source emergency room | 15964 | 7384 | 8580 | 26.08 % | 24.47 % | 27.65 % | Admission source | 965 | 457 | 508 | 0.0157672 | 0.0151470 | 0.0163702 |
| Admission source hospital ward | 15160 | 7571 | 7589 | 24.77 % | 25.09 % | 24.46 % | Admission source | 965 | 457 | 508 | 0.0157672 | 0.0151470 | 0.0163702 |
| Admission source other hospital | 9015 | 4800 | 4215 | 14.73 % | 15.91 % | 13.58 % | Admission source | 965 | 457 | 508 | 0.0157672 | 0.0151470 | 0.0163702 |
| Age | 64.00 | 64.00 | 63.00 | (53.00-74.00) | (53.00-74.00) | (53.00-73.00) | Age | 0 | 0 | 0 | 0.0000000 | 0.0000000 | 0.0000000 |
| Shifted log maximum bilirubin | 0.44 | 0.44 | 0.45 | (0.29-0.69) | (0.29-0.69) | (0.29-0.69) | Bilirubin | 14,165 | 6,920 | 7,245 | 0.2314429 | 0.2293593 | 0.2334687 |
| Shifted log mean bilirubin | 0.44 | 0.44 | 0.44 | (0.29-0.68) | (0.29-0.68) | (0.29-0.68) | Bilirubin | 14,165 | 6,920 | 7,245 | 0.2314429 | 0.2293593 | 0.2334687 |
| Shifted log minimum bilirubin | 0.43 | 0.43 | 0.43 | (0.28-0.67) | (0.29-0.66) | (0.28-0.67) | Bilirubin | 14,165 | 6,920 | 7,245 | 0.2314429 | 0.2293593 | 0.2334687 |
| BMI | 26.42 | 26.57 | 26.30 | (24.10-29.98) | (24.09-30.19) | (24.15-29.67) | BMI | 757 | 348 | 409 | 0.0123687 | 0.0115343 | 0.0131799 |
| Shifted log sd body temperature | 0.28 | 0.28 | 0.27 | (0.19-0.40) | (0.19-0.40) | (0.19-0.39) | Body temperature sd | 2,898 | 1,383 | 1,515 | 0.0473506 | 0.0458387 | 0.0488206 |
| Maximum body temperature | 37.50 | 37.50 | 37.50 | (37.00-38.10) | (37.00-38.10) | (37.00-38.10) | Body temperature | 829 | 365 | 464 | 0.0135451 | 0.0120977 | 0.0149523 |
| Mean body temperature | 36.95 | 36.95 | 36.96 | (36.51-37.46) | (36.50-37.44) | (36.52-37.48) | Body temperature | 829 | 365 | 464 | 0.0135451 | 0.0120977 | 0.0149523 |
| Median body temperature | 37.00 | 37.00 | 37.00 | (36.50-37.50) | (36.50-37.50) | (36.50-37.50) | Body temperature | 829 | 365 | 464 | 0.0135451 | 0.0120977 | 0.0149523 |
| Minimum body temperature | 36.50 | 36.40 | 36.50 | (35.90-37.00) | (35.90-36.90) | (36.00-37.00) | Body temperature | 829 | 365 | 464 | 0.0135451 | 0.0120977 | 0.0149523 |
| Q1 body temperature | 36.80 | 36.77 | 36.80 | (36.30-37.27) | (36.30-37.22) | (36.30-37.30) | Body temperature | 829 | 365 | 464 | 0.0135451 | 0.0120977 | 0.0149523 |
| Q3 body temperature | 37.20 | 37.20 | 37.20 | (36.70-37.70) | (36.70-37.70) | (36.70-37.70) | Body temperature | 829 | 365 | 464 | 0.0135451 | 0.0120977 | 0.0149523 |
| Shifted log max creatinine | 0.68 | 0.69 | 0.68 | (0.51-0.96) | (0.51-0.97) | (0.51-0.96) | Creatinine | 3,644 | 1,899 | 1,745 | 0.0595396 | 0.0629412 | 0.0562323 |
| Shifted log mean creatinine | 0.68 | 0.68 | 0.68 | (0.51-0.96) | (0.51-0.96) | (0.50-0.95) | Creatinine | 3,644 | 1,899 | 1,745 | 0.0595396 | 0.0629412 | 0.0562323 |
| Shifted log min creatinine | 0.67 | 0.68 | 0.67 | (0.50-0.95) | (0.51-0.95) | (0.50-0.94) | Creatinine | 3,644 | 1,899 | 1,745 | 0.0595396 | 0.0629412 | 0.0562323 |
| Maximum CRP | 98.00 | 97.50 | 99.00 | (45.85-172.00) | (46.00-171.00) | (45.27-173.00) | CRP | 3,201 | 1,605 | 1,596 | 0.0523014 | 0.0531968 | 0.0514308 |
| Mean CRP | 96.10 | 95.00 | 98.00 | (45.00-169.00) | (45.00-169.00) | (44.48-170.00) | CRP | 3,201 | 1,605 | 1,596 | 0.0523014 | 0.0531968 | 0.0514308 |
| Minimum CRP | 94.50 | 93.00 | 96.00 | (43.10-167.00) | (43.80-166.00) | (43.00-168.00) | CRP | 3,201 | 1,605 | 1,596 | 0.0523014 | 0.0531968 | 0.0514308 |
| Shifted log dobutamine | 0.00 | 0.00 | 0.00 | (0.00-0.00) | (0.00-0.00) | (0.00-0.00) | Dobutamine | 12 | 8 | 4 | 0.0001961 | 0.0002652 | 0.0001289 |
| Shifted log epinephrine | 0.00 | 0.00 | 0.00 | (0.00-0.00) | (0.00-0.00) | (0.00-0.00) | Epinephrine | 4 | 1 | 3 | 0.0000654 | 0.0000331 | 0.0000967 |
| Fluid balance | 264.57 | 274.95 | 255.76 | (-541.41-1191.89) | (-551.05-1228.32) | (-529.41-1159.65) | Fluid balance period | 15 | 8 | 7 | 0.0002451 | 0.0002652 | 0.0002256 |
| Fluid balance stay | 3161.99 | 3126.79 | 3196.10 | (25.08-9648.64) | (29.44-9635.74) | (18.28-9658.81) | Fluid balance stay | 3,451 | 1,794 | 1,657 | 0.0563861 | 0.0594611 | 0.0533965 |
| Maximum GCS | 15.00 | 15.00 | 15.00 | (12.00-15.00) | (11.00-15.00) | (12.00-15.00) | GCS | 40,145 | 19,755 | 20,390 | 0.6559319 | 0.6547678 | 0.6570637 |
| Mean GCS | 14.50 | 14.50 | 14.50 | (11.00-15.00) | (11.00-15.00) | (11.00-15.00) | GCS | 40,145 | 19,755 | 20,390 | 0.6559319 | 0.6547678 | 0.6570637 |
| Minimun GCS | 15.00 | 15.00 | 15.00 | (10.00-15.00) | (10.00-15.00) | (10.00-15.00) | GCS | 40,145 | 19,755 | 20,390 | 0.6559319 | 0.6547678 | 0.6570637 |
| Maximum Hb | 8.80 | 8.80 | 8.80 | (8.00-10.10) | (8.00-10.10) | (8.00-10.10) | Hb | 2,297 | 1,164 | 1,133 | 0.0375308 | 0.0385801 | 0.0365107 |
| Mean Hb | 8.70 | 8.70 | 8.70 | (8.00-10.00) | (8.00-10.00) | (8.00-10.00) | Hb | 2,297 | 1,164 | 1,133 | 0.0375308 | 0.0385801 | 0.0365107 |
| Minimum Hb | 8.70 | 8.70 | 8.70 | (7.90-9.90) | (7.90-9.90) | (7.90-9.90) | Hb | 2,297 | 1,164 | 1,133 | 0.0375308 | 0.0385801 | 0.0365107 |
| Maximum HR | 108.00 | 109.00 | 108.00 | (94.00-123.00) | (94.00-124.00) | (94.00-123.00) | HR | 1 | 1 | 0 | 0.0000163 | 0.0000331 | 0.0000000 |
| Mean HR | 87.30 | 87.54 | 87.06 | (75.48-99.96) | (75.62-100.54) | (75.37-99.49) | HR | 1 | 1 | 0 | 0.0000163 | 0.0000331 | 0.0000000 |
| Median HR | 86.50 | 87.00 | 86.00 | (74.50-100.00) | (74.75-100.00) | (74.00-99.00) | HR | 1 | 1 | 0 | 0.0000163 | 0.0000331 | 0.0000000 |
| Minimum HR | 71.00 | 71.00 | 71.00 | (60.00-83.00) | (61.00-83.00) | (60.00-83.00) | HR | 1 | 1 | 0 | 0.0000163 | 0.0000331 | 0.0000000 |
| Q1 HR | 81.00 | 81.00 | 80.75 | (69.00-93.50) | (69.50-94.00) | (69.00-93.00) | HR | 1 | 1 | 0 | 0.0000163 | 0.0000331 | 0.0000000 |
| Q3 HR | 93.00 | 93.00 | 92.25 | (80.00-106.25) | (80.00-107.00) | (80.00-106.00) | HR | 1 | 1 | 0 | 0.0000163 | 0.0000331 | 0.0000000 |
| SD HR | 7.83 | 7.85 | 7.81 | (5.65-10.85) | (5.64-10.88) | (5.66-10.81) | HR sd | 3 | 2 | 1 | 0.0000490 | 0.0000663 | 0.0000322 |
| Shifted log max lactate | 0.92 | 0.92 | 0.92 | (0.74-1.13) | (0.74-1.13) | (0.74-1.13) | Lactate | 1,176 | 587 | 589 | 0.0192147 | 0.0194558 | 0.0189804 |
| Shifted log mean lactate | 0.76 | 0.77 | 0.75 | (0.62-0.94) | (0.63-0.95) | (0.62-0.93) | Lactate | 1,176 | 587 | 589 | 0.0192147 | 0.0194558 | 0.0189804 |
| Shifted log min lactate | 0.59 | 0.59 | 0.59 | (0.47-0.74) | (0.47-0.79) | (0.47-0.74) | Lactate | 1,176 | 587 | 589 | 0.0192147 | 0.0194558 | 0.0189804 |
| Maximum leukocytes | 11.36 | 11.44 | 11.26 | (8.24-15.38) | (8.34-15.59) | (8.15-15.19) | Leukocytes | 2,387 | 1,209 | 1,178 | 0.0390014 | 0.0400716 | 0.0379608 |
| Mean leukocytes | 11.15 | 11.26 | 11.04 | (8.15-15.09) | (8.25-15.29) | (8.07-14.85) | Leukocytes | 2,387 | 1,209 | 1,178 | 0.0390014 | 0.0400716 | 0.0379608 |
| Minimum leukocytes | 10.92 | 11.02 | 10.82 | (7.99-14.81) | (8.08-15.03) | (7.91-14.58) | Leukocytes | 2,387 | 1,209 | 1,178 | 0.0390014 | 0.0400716 | 0.0379608 |
| Maximum MAP | 110.00 | 109.00 | 110.00 | (98.00-124.00) | (98.00-124.00) | (99.00-125.00) | MAP | 15 | 4 | 11 | 0.0002451 | 0.0001326 | 0.0003545 |
| Mean MAP | 81.22 | 80.67 | 81.72 | (75.23-90.19) | (74.93-89.44) | (75.53-90.85) | MAP | 15 | 4 | 11 | 0.0002451 | 0.0001326 | 0.0003545 |
| Median MAP | 80.50 | 80.00 | 81.00 | (74.00-90.00) | (74.00-89.00) | (74.50-91.00) | MAP | 15 | 4 | 11 | 0.0002451 | 0.0001326 | 0.0003545 |
| Minimum MAP | 61.00 | 61.00 | 61.00 | (55.00-69.00) | (55.00-68.00) | (55.00-69.00) | MAP | 15 | 4 | 11 | 0.0002451 | 0.0001326 | 0.0003545 |
| Q1 MAP | 74.00 | 74.00 | 74.75 | (69.00-83.00) | (68.75-82.00) | (69.00-83.50) | MAP | 15 | 4 | 11 | 0.0002451 | 0.0001326 | 0.0003545 |
| Q3 MAP | 87.25 | 87.00 | 88.00 | (80.00-97.50) | (80.00-97.00) | (80.75-98.00) | MAP | 15 | 4 | 11 | 0.0002451 | 0.0001326 | 0.0003545 |
| SD MAP | 9.96 | 9.91 | 10.00 | (7.90-12.47) | (7.86-12.44) | (7.94-12.48) | MAP sd | 24 | 11 | 13 | 0.0003921 | 0.0003646 | 0.0004189 |
| Shifted log norepinephrine | 0.73 | 0.83 | 0.64 | (0.00-2.31) | (0.00-2.38) | (0.00-2.25) | Norepinephrine | 61 | 30 | 31 | 0.0009967 | 0.0009943 | 0.0009990 |
| Maximum O_2_ saturation | 100.00 | 100.00 | 100.00 | (99.10-100.00) | (99.00-100.00) | (99.10-100.00) | O_2_ saturation | 8 | 4 | 4 | 0.0001307 | 0.0001326 | 0.0001289 |
| Mean O_2_ saturation | 97.22 | 97.17 | 97.25 | (95.88-98.34) | (95.85-98.30) | (95.90-98.37) | O_2_ saturation | 8 | 4 | 4 | 0.0001307 | 0.0001326 | 0.0001289 |
| Median O_2_ saturation | 97.50 | 97.40 | 97.60 | (96.00-99.00) | (96.00-98.90) | (96.00-99.00) | O_2_ saturation | 8 | 4 | 4 | 0.0001307 | 0.0001326 | 0.0001289 |
| Minimum O_2_ saturation | 93.00 | 93.00 | 93.00 | (90.00-95.00) | (90.00-95.00) | (90.00-95.00) | O_2_ saturation | 8 | 4 | 4 | 0.0001307 | 0.0001326 | 0.0001289 |
| Q1 O_2_ saturation | 96.08 | 96.00 | 96.20 | (95.00-97.83) | (94.90-97.75) | (95.00-97.90) | O_2_ saturation | 8 | 4 | 4 | 0.0001307 | 0.0001326 | 0.0001289 |
| Q3 O_2_ saturation | 98.55 | 98.50 | 98.65 | (97.10-100.00) | (97.03-100.00) | (97.15-100.00) | O_2_ saturation | 8 | 4 | 4 | 0.0001307 | 0.0001326 | 0.0001289 |
| Shifted log sd O_2_ saturation | 1.00 | 1.00 | 1.00 | (0.81-1.21) | (0.81-1.21) | (0.80-1.20) | O_2_ saturation sd | 31 | 16 | 15 | 0.0005065 | 0.0005303 | 0.0004834 |
| Maximum paO_2_/FiO_2_ | 336.67 | 337.14 | 336.67 | (276.67-393.33) | (275.00-390.00) | (277.76-396.67) | paO_2_/FiO_2_ | 17,824 | 8,795 | 9,029 | 0.2912276 | 0.2915051 | 0.2909577 |
| Mean paO_2_/FiO_2_ | 274.99 | 274.55 | 275.18 | (225.04-326.25) | (222.75-322.77) | (227.47-329.29) | paO_2_/FiO_2_ | 17,824 | 8,795 | 9,029 | 0.2912276 | 0.2915051 | 0.2909577 |
| Minimum paO_2_/FiO_2_ | 213.33 | 213.33 | 213.33 | (170.00-270.00) | (169.00-266.67) | (171.11-270.33) | paO_2_/FiO_2_ | 17,824 | 8,795 | 9,029 | 0.2912276 | 0.2915051 | 0.2909577 |
| Maximum PEEP | 8.00 | 8.00 | 8.00 | (6.00-10.00) | (6.00-10.00) | (6.00-9.80) | PEEP | 17,233 | 8,467 | 8,766 | 0.2815712 | 0.2806337 | 0.2824826 |
| Mean PEEP | 7.65 | 7.65 | 7.65 | (6.00-8.52) | (6.00-8.68) | (6.00-8.15) | PEEP | 17,233 | 8,467 | 8,766 | 0.2815712 | 0.2806337 | 0.2824826 |
| Median PEEP | 8.00 | 8.00 | 7.84 | (6.00-8.00) | (6.00-8.00) | (6.00-8.00) | PEEP | 17,233 | 8,467 | 8,766 | 0.2815712 | 0.2806337 | 0.2824826 |
| Minimum PEEP | 6.00 | 6.00 | 6.00 | (5.88-8.00) | (5.88-8.00) | (5.88-8.00) | PEEP | 17,233 | 8,467 | 8,766 | 0.2815712 | 0.2806337 | 0.2824826 |
| Q1 PEEP | 7.00 | 7.00 | 7.00 | (6.00-8.00) | (6.00-8.00) | (6.00-8.00) | PEEP | 17,233 | 8,467 | 8,766 | 0.2815712 | 0.2806337 | 0.2824826 |
| Q3 PEEP | 8.00 | 8.00 | 8.00 | (6.00-9.00) | (6.00-9.80) | (6.00-8.00) | PEEP | 17,233 | 8,467 | 8,766 | 0.2815712 | 0.2806337 | 0.2824826 |
| Shifted log sd PEEP | 0.00 | 0.00 | 0.00 | (0.00-0.40) | (0.00-0.40) | (0.00-0.40) | PEEP sd | 17,472 | 8,590 | 8,882 | 0.2854762 | 0.2847105 | 0.2862207 |
| Maximum pH | 7.46 | 7.46 | 7.46 | (7.42-7.49) | (7.42-7.49) | (7.42-7.49) | pH | 2,602 | 1,281 | 1,321 | 0.0425143 | 0.0424580 | 0.0425690 |
| Mean pH | 7.42 | 7.42 | 7.42 | (7.38-7.45) | (7.38-7.45) | (7.38-7.45) | pH | 2,602 | 1,281 | 1,321 | 0.0425143 | 0.0424580 | 0.0425690 |
| Minimum pH | 7.39 | 7.39 | 7.39 | (7.33-7.43) | (7.33-7.43) | (7.34-7.43) | pH | 2,602 | 1,281 | 1,321 | 0.0425143 | 0.0424580 | 0.0425690 |
| Maximum RASS | -2.00 | -2.00 | -2.00 | (-4.00-0.00) | (-4.00--1.00) | (-4.00-0.00) | RASS | 3,949 | 2,005 | 1,944 | 0.0645230 | 0.0664545 | 0.0626450 |
| Maximum RASS | 0.00 | 0.00 | 0.00 | (-3.00-1.00) | (-3.00-1.00) | (-3.00-1.00) | RASS | 3,949 | 2,005 | 1,944 | 0.0645230 | 0.0664545 | 0.0626450 |
| Mean RASS | -1.00 | -1.00 | -0.88 | (-3.60-0.00) | (-3.67-0.00) | (-3.50-0.00) | RASS | 3,949 | 2,005 | 1,944 | 0.0645230 | 0.0664545 | 0.0626450 |
| Renal replacement  therapy | 12141 | 6410 | 5731 | 19.84 % | 21.25 % | 18.47 % | Renal replacement | 0 | 0 | 0 | 0.0000000 | 0.0000000 | 0.0000000 |
| Maximum RR | 28.00 | 28.00 | 28.00 | (24.00-33.00) | (24.00-33.00) | (24.00-33.00) | RR | 386 | 180 | 206 | 0.0063069 | 0.0059660 | 0.0066383 |
| Mean RR | 19.24 | 19.25 | 19.22 | (16.54-22.17) | (16.57-22.20) | (16.51-22.14) | RR | 386 | 180 | 206 | 0.0063069 | 0.0059660 | 0.0066383 |
| Median RR | 19.00 | 19.00 | 19.00 | (16.00-22.00) | (16.00-22.00) | (16.00-22.00) | RR | 386 | 180 | 206 | 0.0063069 | 0.0059660 | 0.0066383 |
| Minimum RR | 12.00 | 12.00 | 12.00 | (9.00-14.00) | (9.00-14.00) | (9.00-14.00) | RR | 386 | 180 | 206 | 0.0063069 | 0.0059660 | 0.0066383 |
| Q1 RR | 17.00 | 17.00 | 17.00 | (14.00-20.00) | (14.00-20.00) | (14.00-20.00) | RR | 386 | 180 | 206 | 0.0063069 | 0.0059660 | 0.0066383 |
| Q3 RR | 21.00 | 21.00 | 21.00 | (18.00-24.00) | (18.00-24.00) | (18.00-24.00) | RR | 386 | 180 | 206 | 0.0063069 | 0.0059660 | 0.0066383 |
| SD RR | 3.32 | 3.32 | 3.31 | (2.48-4.30) | (2.48-4.29) | (2.48-4.30) | RR sd | 414 | 198 | 216 | 0.0067644 | 0.0065626 | 0.0069606 |
| Sex male | 1.00 | 1.00 | 1.00 | (0.00-1.00) | (0.00-1.00) | (0.00-1.00) | Sex male | 103 | 41 | 62 | 0.0016829 | 0.0013589 | 0.0019979 |
| Maximum thrombocytes | 219.00 | 215.00 | 223.00 | (143.00-312.00) | (138.00-305.00) | (148.00-320.00) | Thrombocytes | 2,508 | 1,308 | 1,200 | 0.0409784 | 0.0433529 | 0.0386698 |
| Mean thrombocytes | 216.00 | 211.50 | 220.00 | (141.00-310.00) | (136.00-302.00) | (146.00-317.00) | Thrombocytes | 2,508 | 1,308 | 1,200 | 0.0409784 | 0.0433529 | 0.0386698 |
| Minimum thrombocytes | 214.00 | 209.00 | 216.00 | (139.00-307.00) | (134.00-300.00) | (143.00-315.00) | Thrombocytes | 2,508 | 1,308 | 1,200 | 0.0409784 | 0.0433529 | 0.0386698 |
| Maximum tidal volumne | 1.58 | 1.58 | 1.58 | (1.40-1.82) | (1.38-1.81) | (1.42-1.82) | Tidal volume | 23,127 | 11,422 | 11,705 | 0.3778736 | 0.3785755 | 0.3771913 |
| Mean tidal volumne | 1.12 | 1.11 | 1.12 | (1.05-1.23) | (1.04-1.22) | (1.06-1.24) | Tidal volume | 23,127 | 11,422 | 11,705 | 0.3778736 | 0.3785755 | 0.3771913 |
| Median tidal volumne | 1.10 | 1.10 | 1.10 | (1.05-1.23) | (1.04-1.22) | (1.06-1.23) | Tidal volume | 23,127 | 11,422 | 11,705 | 0.3778736 | 0.3785755 | 0.3771913 |
| Minimum tidal volumne | 0.62 | 0.62 | 0.62 | (0.41-0.77) | (0.41-0.77) | (0.42-0.77) | Tidal volume | 23,127 | 11,422 | 11,705 | 0.3778736 | 0.3785755 | 0.3771913 |
| Q1 tidal volumne | 1.03 | 1.03 | 1.03 | (0.96-1.11) | (0.95-1.11) | (0.97-1.12) | Tidal volume | 23,127 | 11,422 | 11,705 | 0.3778736 | 0.3785755 | 0.3771913 |
| Q3 tidal volumne | 1.19 | 1.19 | 1.20 | (1.13-1.36) | (1.12-1.35) | (1.14-1.36) | Tidal volume | 23,127 | 11,422 | 11,705 | 0.3778736 | 0.3785755 | 0.3771913 |
| SD tidal volumne | 0.21 | 0.21 | 0.21 | (0.16-0.27) | (0.16-0.27) | (0.16-0.27) | Tidal volume sd | 23,479 | 11,584 | 11,895 | 0.3836250 | 0.3839448 | 0.3833140 |
| Shifted log ventilator use | 3.89 | 3.89 | 3.89 | (0.00-5.25) | (0.00-5.25) | (0.00-5.24) | Ventilator use | 45 | 4 | 41 | 0.0007353 | 0.0001326 | 0.0013212 |

Listed are all features (97 features) used to calculate the machine learning algorithms, excluding missing feature indicators (34 features). Invalid value percentages are based on the number of available measurements.

BMI, body mass index. CRP, C-reactive protein. GCS, Glasgow Coma Scale. Hb, hemoglobin. HR, heart rate. ICU, intensive care unit. IQR, interquartile range. PEEP, Positive end-expiratory pressure. MAP, mean arterial pressure. Min, minimum. Max, maximum. Q1, 1^st^ quartile. Q3, 3^rd^ quartile. RASS, Richmond Agitation-Sedation Scale. RR, respiratory rate. Sd, standard deviation. Shifted log, shifted logarithm transformed.

Supplemental Table 4**:** *Variables used for the LGBM-48h algorithm, SAPS II/TISS-10, and SOFA score.*

|  | **Machine learning algorithms** | **SAPS II/TISS-10** | **SOFA score** |
| --- | --- | --- | --- |
| **Vital signs** |  |  |  |
| Blood pressure, systolic/diastolic |  | X |  |
| Blood pressure, MAP | X |  | X |
| Heart rate | X | X |  |
| O_2_ saturation | X |  |  |
| Respiratory rate | X |  | X |
| Body temperature | X | X |  |
| Glasgow coma scale | X |  | X |
| RASS | X |  |  |
| Daily fluid balance | X | X |  |
| **Labaratory parameters** |  |  |  |
| Lactate | X |  |  |
| pH | X |  |  |
| Bicarbonate |  | X |  |
| Potassium |  | X |  |
| Sodium |  | X |  |
| Hemoglobin | X |  |  |
| Thrombocytes | X |  | X |
| Leukocytes | X | X |  |
| C-reactive protein | X |  |  |
| Creatinine | X |  | X |
| Blood urea nitrogen |  | X |  |
| Bilirubin | X | X | X |
| **Medication** |  |  |  |
| Catecholamine use | X^1^ | X^2^ | X^3^ |
| **Ventilation** |  |  |  |
| NIV/IMV | X | X | X |
| PaO_2_/F_i_O_2_ | X | X | X |
| PEEP | X |  |  |
| Tidal volume | X |  |  |
| **Interventions** |  |  |  |
| Renal replacement therapy | X | X |  |
| Arterial line |  | X |  |
| Pulmonary catheter |  | X |  |
| Intercerebral catheter |  | X |  |
| Postpyloric tube |  | X |  |
| Special interventions |  | X^4^ |  |
| Metabolic therapy |  | X |  |
| Patient position therapy |  | X |  |
| Intrahospital transport |  | X |  |
| **Demographics** |  |  |  |
| Age | X | X |  |
| Sex | X |  |  |
| Body mass index | X |  |  |
| Chronic disease |  | X |  |
| Admission source | X | X^5^ |  |

IMV, invasive mechanical ventilation. MAP, mean arterial pressure. NIV, noninvasive ventilation. RASS, Richmond Agitation-Sedation Scale. SAPS II, Simplified Acute Physiology Score II. SOFA score, Sequential Organ Failure Assessment score. TISS-10 Therapeutic Intervention Scoring System.

^1^Norepinephrine, adrenaline/epinephrine, dobutamine.

^2^Noradrenaline, adrenaline/epinephrine, dobutamine, dopamine, milrinone, levosimendan, vasopressin. ^3^Norepinephrine, adrenaline/epinephrine, dobutamine.

^4^Interventions: intubation, tracheotomy, resuscitation, defibrillation, bronchoscopy, punctions in body cavities, drainage systems.

^5^Medical, planned surgical, unplanned surgical.

Supplemental Table 5: *Patient and stay days with and without difference features for the training and test dataset.*

|  | **Training set** | **Test set** |
| --- | --- | --- |
| **Patient stays** | | |
| With difference features | 3,345 | 3,320 |
| Without difference features | 4,892 | 4,894 |
| **Stay days** | | |
| With differences features | 26,140 | 25,277 |
| Without differences features | 31,032 | 30,171 |

Supplemental Table 6: *Performance of the different machine learning algorithms with data from the training dataset.*

| **Machine learning algorithm** | **AUROC** | **95% confidence interval** |
| --- | --- | --- |
| Light Gradient-Boosting Machine | 0.909 | 0.901 - 0.917 |
| Extreme Gradient Boosting | 0.902 | 0.894 - 0.911 |
| Random Forest | 0.899 | 0.890 - 0.908 |
| Equivariant Implicit Neural Network | 0.872 | 0.861 - 0.882 |
| Least Absolute Shrinkage and Selection Operator | 0.871 | 0.860 - 0.882 |

AUROC, area under the receiver operating characteristics curve.

Supplemental Table 7: *Youden´s index, F1-score sensitivity, specificity and alarm rates.*

| **Threshold** | **Sensitivity** | **Specificity** | **Alarm rate** | **Youden´s index** | **F1-score** |
| --- | --- | --- | --- | --- | --- |
| 0.001 | 0.99857347 | 0.1495360 | 0.857346459 | 0.14810943 | 0.10268070 |
| 0.002 | 0.99144080 | 0.2721680 | 0.740081535 | 0.26360876 | 0.11714635 |
| 0.003 | 0.98145506 | 0.3508638 | 0.664578569 | 0.33231884 | 0.12828043 |
| 0.004 | 0.97717546 | 0.4087386 | 0.609194259 | 0.38591404 | 0.13850976 |
| 0.005 | 0.96504993 | 0.4560117 | 0.563554407 | 0.42106161 | 0.14702526 |
| 0.006 | 0.96005706 | 0.4923007 | 0.528719631 | 0.45235780 | 0.15512274 |
| 0.007 | 0.95435093 | 0.5243491 | 0.497895330 | 0.47870005 | 0.16293229 |
| 0.008 | 0.94721826 | 0.5516354 | 0.471545524 | 0.49885370 | 0.16994050 |
| 0.009 | 0.93937233 | 0.5755501 | 0.448377581 | 0.51492240 | 0.17642331 |
| 0.010 | 0.93223966 | 0.5948764 | 0.429617845 | 0.52711609 | 0.18198273 |
| 0.011 | 0.92368046 | 0.6136466 | 0.411322131 | 0.53732709 | 0.18751810 |
| 0.012 | 0.91440799 | 0.6308179 | 0.394517915 | 0.54522588 | 0.19270951 |
| 0.013 | 0.90798859 | 0.6473287 | 0.378476020 | 0.55531731 | 0.19858045 |
| 0.014 | 0.90299572 | 0.6613716 | 0.364853667 | 0.56436734 | 0.20402901 |
| 0.015 | 0.89728959 | 0.6747541 | 0.351827914 | 0.57204366 | 0.20937006 |
| 0.016 | 0.89300999 | 0.6868157 | 0.340127937 | 0.57982566 | 0.21467764 |
| 0.017 | 0.88231098 | 0.6986339 | 0.328361672 | 0.58094493 | 0.21876382 |
| 0.018 | 0.87446505 | 0.7090966 | 0.318020616 | 0.58356165 | 0.22296990 |
| 0.019 | 0.86733238 | 0.7185165 | 0.308707037 | 0.58584884 | 0.22695035 |
| 0.020 | 0.86091298 | 0.7276930 | 0.299658613 | 0.58860598 | 0.23115963 |
| 0.021 | 0.85734665 | 0.7371476 | 0.290477611 | 0.59449427 | 0.23647452 |
| 0.022 | 0.84807418 | 0.7454552 | 0.282125220 | 0.59352936 | 0.23986282 |
| 0.023 | 0.84022825 | 0.7531718 | 0.274402572 | 0.59340006 | 0.24336329 |
| 0.024 | 0.83166904 | 0.7604018 | 0.267110802 | 0.59207087 | 0.24648557 |
| 0.025 | 0.82239658 | 0.7667281 | 0.260647642 | 0.58912465 | 0.24886682 |
| 0.026 | 0.81455064 | 0.7733324 | 0.253985615 | 0.58788305 | 0.25195808 |
| 0.027 | 0.80813124 | 0.7794501 | 0.247853899 | 0.58758134 | 0.25518018 |
| 0.028 | 0.80099857 | 0.7859154 | 0.241357595 | 0.58691397 | 0.25863657 |
| 0.029 | 0.79671897 | 0.7918941 | 0.235457890 | 0.58861303 | 0.26263814 |
| 0.030 | 0.79101284 | 0.7974904 | 0.229856485 | 0.58850319 | 0.26604294 |
| 0.040 | 0.74322397 | 0.8416351 | 0.185542408 | 0.58485906 | 0.29771429 |
| 0.050 | 0.70328103 | 0.8712155 | 0.155480428 | 0.57449657 | 0.32365009 |
| 0.060 | 0.66975749 | 0.8915499 | 0.134533161 | 0.56130742 | 0.34389306 |
| 0.070 | 0.63980029 | 0.9064966 | 0.118888999 | 0.54629686 | 0.35959110 |
| 0.080 | 0.61198288 | 0.9198443 | 0.104868914 | 0.53182716 | 0.37582129 |
| 0.090 | 0.59272468 | 0.9304112 | 0.093898114 | 0.52313589 | 0.39244392 |
| 0.100 | 0.57346648 | 0.9373979 | 0.086341189 | 0.51086437 | 0.40129773 |
| 0.110 | 0.55278174 | 0.9443151 | 0.078784263 | 0.49709680 | 0.41016142 |
| 0.120 | 0.53922967 | 0.9498071 | 0.072917702 | 0.48903676 | 0.41976680 |
| 0.130 | 0.51997147 | 0.9541520 | 0.067879752 | 0.47412351 | 0.42260870 |
| 0.140 | 0.50427960 | 0.9587403 | 0.062775513 | 0.46301991 | 0.42900485 |
| 0.150 | 0.49429387 | 0.9617992 | 0.059394783 | 0.45609302 | 0.43393863 |
| 0.160 | 0.48074180 | 0.9650318 | 0.055682609 | 0.44577360 | 0.43737833 |
| 0.170 | 0.46718973 | 0.9675693 | 0.052633323 | 0.43475899 | 0.43812709 |
| 0.180 | 0.45720399 | 0.9698634 | 0.049981771 | 0.42706739 | 0.44054983 |
| 0.190 | 0.44793153 | 0.9720880 | 0.047429651 | 0.42001954 | 0.44334628 |
| 0.200 | 0.43723252 | 0.9742083 | 0.044910676 | 0.41144087 | 0.44468625 |
| 0.300 | 0.34308131 | 0.9858876 | 0.029399092 | 0.32896890 | 0.42027086 |
| 0.400 | 0.28958631 | 0.9919705 | 0.021112989 | 0.28155683 | 0.39823443 |
| 0.500 | 0.23109843 | 0.9956203 | 0.014914985 | 0.22671872 | 0.34989201 |
| 0.600 | 0.18188302 | 0.9972887 | 0.011037089 | 0.17917177 | 0.29394813 |
| 0.700 | 0.13124108 | 0.9984011 | 0.007623214 | 0.12964214 | 0.22549020 |
| 0.800 | 0.08630528 | 0.9993396 | 0.004640217 | 0.08564485 | 0.15693904 |
| 0.900 | 0.04208274 | 0.9996176 | 0.002320109 | 0.04170038 | 0.08016304 |

Values highlighted in yellow represent the maximum F1-score and the maximum Youden’s index, respectively.

Supplemental Table 8: *Predictive performance at the low and high threshold comparing the University Medical Center Mannheim ICU dataset and the MIMIC-IV dataset.*

|  | **University Medical Center Mannheim ICU dataset** | | **MIMIC-IV dataset** | |
| --- | --- | --- | --- | --- |
| Threshold | **Low threshold** (2.1% predicted 48-hour mortality) | **High threshold**  (20.0% predicted 48-hour mortality) | **Low threshold** (2.1% predicted 48-hour mortality) | **High threshold** (20.0% predicted 48-hour mortality) |
| Sensitivity | 0.857 | 0.437 | 0.766 | 0.346 |
| Specificity | 0.737 | 0.974 | 0.784 | 0.977 |
| Positive predictive value (PPV) | 0.137 | 0.452 | 0.100 | 0.320 |
| Negative predictive value (NPV) | 0.991 | 0.973 | 0.991 | 0.979 |
| Stay days below threshold,  n (%) | 21,407 (70.9) | 28,816 (95.5) | 30,131 (76.7) | 37,965 (96.7) |
| Stay days above threshold,  n (%) | 8,764 (29.1) | 1,355 (4.5) | 9,128 (23.3) | 1,294 (3.3) |

Supplemental Table 9: *Distribution of risk group categories for patient stays.*

|  | **No change in risk category** | | | **Change across two mortality risk categories** | | | **Change across all mortality risk categories** |
| --- | --- | --- | --- | --- | --- | --- | --- |
| **Predicted 48-h mortality risk category** | low | inter-mediate | high | low/  inter-mediate | low/  high | inter-mediate/  high | low/inter-  mediate/ high |
| **Distribution** |  |  |  |  |  |  |  |
| Stay days, n (%) | 9,389  (31.1) | 646  (2.1) | 233  (0.8) | 12,000  (39.8) | 119  (0.4) | 1,105  (3.7) | 6,679  (22.1) |
| Patient stays, n (%) | 2,857  (58.3) | 334  (6.8) | 162  (3.3) | 1,000  (20.5) | 24  (0.5) | 184  (3.7) | 333  (6.9) |
| Mean length of stay, days | 3.8 | 2.5 | 1.9 | 12.6 | 5.4 | 6.4 | 20.7 |
| **ICU mortality** |  |  |  |  |  |  |  |
| Mortality, % | 1.0 | 32.6 | 88.8 | 14.1 | 33.3 | 88.0 | 58.5 |
| **Predictive performance (ICU mortality)** | | | | | | | |
| Sensitivity | 0.311 | 0.945 | 0.995 | 0.791 | 0.996 | 0.994 | 0.966 |
| Specificity | 0.036 | 0.138 | 0.183 | 0.179 | 0.010 | 0.205 | 0.247 |
| Positive predictive value | 0.627 | 0.851 | 0.864 | 0.834 | 0.839 | 0.867 | 0.869 |
| Negative predictive value | 0.010 | 0.326 | 0.888 | 0.141 | 0.333 | 0.880 | 0.585 |
| **In-hospital mortality** |  |  |  |  |  |  |  |
| Mortality, % | 9.9 | 59.3 | 95.6 | 29.9 | 45.8 | 94.5 | 67.5 |
| **Predictive performance (In-hospital mortality)** | | | | | | | |
| Sensitivity | 0.274 | 0.961 | 0.998 | 0.802 | 0.996 | 0.997 | 0.969 |
| Specificity | 0.211 | 0.147 | 0.115 | 0.222 | 0.008 | 0.129 | 0.167 |
| Positive predictive value | 0.478 | 0.748 | 0.748 | 0.731 | 0.729 | 0.751 | 0.754 |
| Negative predictive value | 0.099 | 0.592 | 0.956 | 0.299 | 0.458 | 0.945 | 0.675 |

Supplemental Table 10: *Patient inclusion and exclusion criteria for the final study cohort.*

| **Patient inclusion and exclusion criteria for the final study cohort** |
| --- |
| **Inclusion criteria** |
| ICU admission between Jan 2018 – May 2022 |
| Age ≥ 18 years on admission |
| ICU stay ≥ 24 hours |
| **Exclusion criteria** |
| > 30% missing features during the first 24h |

Supplemental Table 11: *Baseline characteristics of all MIMIC-IV patients included for external validation.*

|  | **MIMIC-IV dataset**  n=10,246 |
| --- | --- |
| **Demographics and medical history** | |
| Age (years), median (IQR) | 66.2 (55.1-76.1) |
| Sex, No. (%) |  |
| Female | 4,133 (40.3) |
| Male | 6,113 (59.7) |
| BMI (kg/m^2^), median (IQR) | 28.1 (24.3-33.0) |
| Diabetes, No. (%) | 2,920 (28.5) |
| Hypercholesterolemia, No. (%) | 4,945 (48.3) |
| Arterial hypertension, No. (%) | 4,039 (39.7) |
| Chronic kidney disease (any stage), No. (%) | 3,393 (33.1) |
| Renal replacement therapy, No. (%) | 638 (6.2) |
| NIV/IMV, No. (%) | 5,246 (51.2) |
| **Main diagnosis^1^, No. (%)** | |
| Diseases of the digestive system | 722 (7.0) |
| Diseases of the nervous system | 350 (3.4) |
| Injury, poisoning and certain other consequences of external causes | 1,255 (12.2) |
| Certain infectious and parasitic diseases | 1,215 (11.8) |
| Diseases of the circulatory system | 4,275 (41.7) |
| Neoplasms | 899 (8.8) |
| Diseases of the respiratory system | 405 (3.9) |
| Diseases of the genitourinary system | 74 (0.7) |
| Other | 1,051 (10.2) |
| **Admission category, No. (%)** | |
| Medical | 760 (7.4) |
| Surgical | 2,625 (25.6) |
| Other | 6,861 (66.9) |
| **Admission source, No. (%)** | |
| Emergency room | 2,975 (29.0) |
| Other ICU | 3,914 (38.2) |
| Hospital ward | 2,223 (21.7) |
| Other | 1,134 (11.0) |
| **Length of ICU stay** | |
| 1 day, No. (%) | 3,696 (36.1) |
| 2 days, No. (%) | 2,080 (20.3) |
| ≥ 3 days, No. (%) | 4,470 (43.6) |
| Length of ICU stay (days), median (IQR) | 2.6 (1.7-4.9) |
| **Mortality, No. (%)** | |
| ICU mortality | 688 (6.7) |
| In-hospital mortality | 1,475 (14.4) |

BMI, body mass index. ICU, intensive care unit. IQR, interquartile range. IMV, invasive mechanical ventilation. NIV, noninvasive ventilation.

^1^According to ICD-10 code, International Classification of Diseases codes, 10th revision, German modification.

Supplemental Table 12: *Optimal cutoff values for 48-hour mortality risk stratification in the MIMIC-IV cohort.*

|  | **MIMIC-IV dataset** | |
| --- | --- | --- |
|  | **Low threshold**  (1.8% predicted 48-hour mortality) | **High threshold**  (19.0% predicted 48-hour mortality) |
| Sensitivity | 0.796 | 0.359 |
| Specificity | 0.759 | 0.975 |
| Positive predictive value (PPV) | 0.094 | 0.314 |
| Negative predictive value (NPV) | 0.992 | 0.980 |
| Stay days below threshold, n (%) | 29,121 (74.2) | 37,893 (96.5) |
| Stay days above threshold, n (%) | 10,138(25.8) | 1,366 (3.5) |

Supplemental Figure 1: *Calibration plot for the LGBM-48h algorithm (shaded area represents 95% confidence band).*

LGBM-48h

*
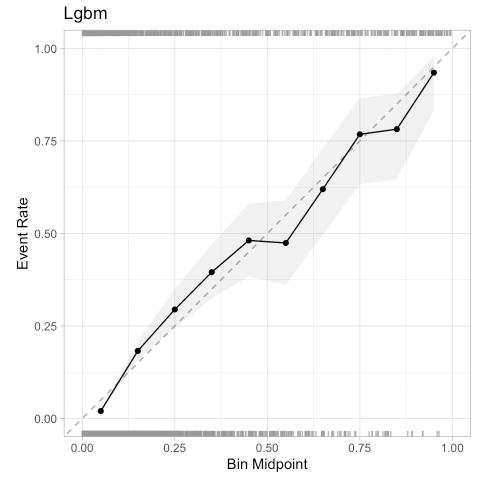
*

Supplemental Figure 2: *Predicted risk distribution and alarm patterns for ICU patients.*


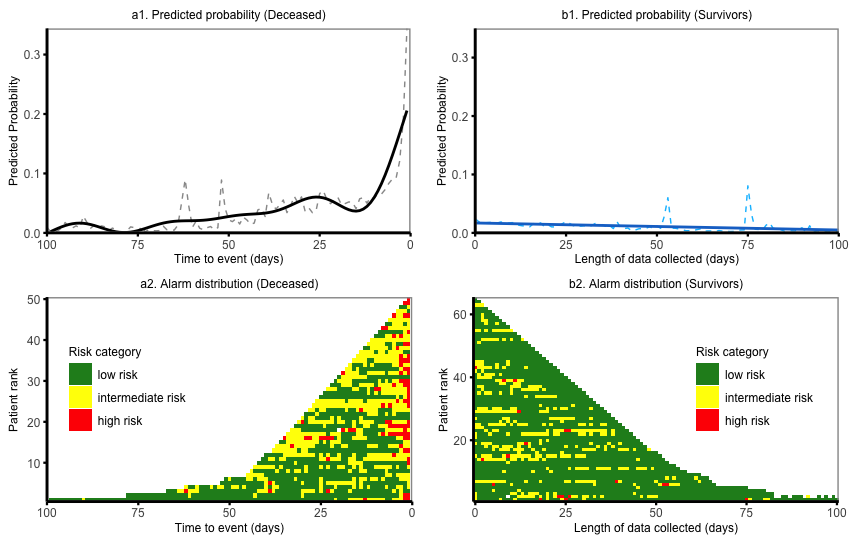


**Supplemental Figure 2: a1, b1:** GAM-smoothed and average predicted probabilities of death over time for ICU patients, stratified by outcome (a1 = deceased, b1 = survivors). The x-axis shows time to event or data collection duration in days; the y-axis indicates the predicted probability of death.
**a2, b2:** Alarm distributions based on risk categories derived from predicted probabilities (green: low risk, yellow: intermediate risk, red: high risk). Patient rank positions reflecting grouped ICU stays, sorted by maximum length of stay (longest stays at the top). a2 shows deceased patients; b2 shows survivors.

Supplemental Figure 3: *Association between risk group exposure and observed ICU mortality across patient stays.*

*
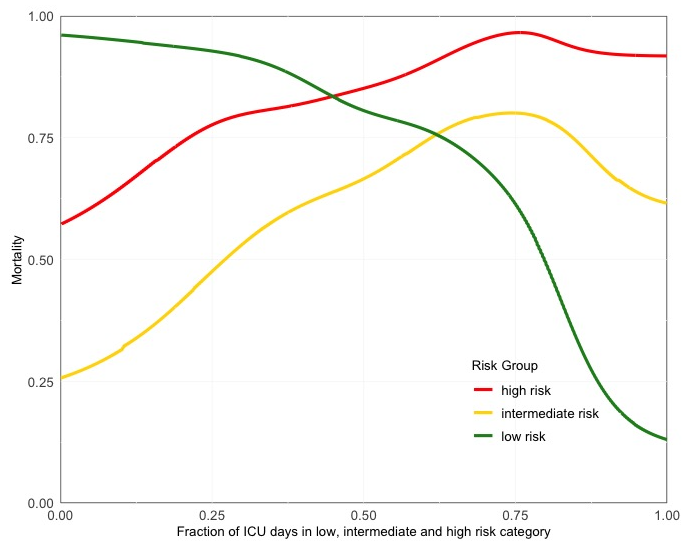
*

**Supplemental Figure 3:** Line plot illustrating ICU mortality based on the fraction of ICU days spent in low-, intermediate-, and high-risk categories per patient stay. Mortality was derived from weighted kernel density estimates stratified by risk group, visualized using generalized additive models (GAM). The curves demonstrate a graded association between time spent in each risk category and observed ICU mortality.

Supplemental Figure 4: *Flow chart illustrating data inclusion from the MIMIC-IV dataset based on patient stays and stay days.*

**a**, Flow chart for ICU patient stays in the MIMIC-IV dataset.


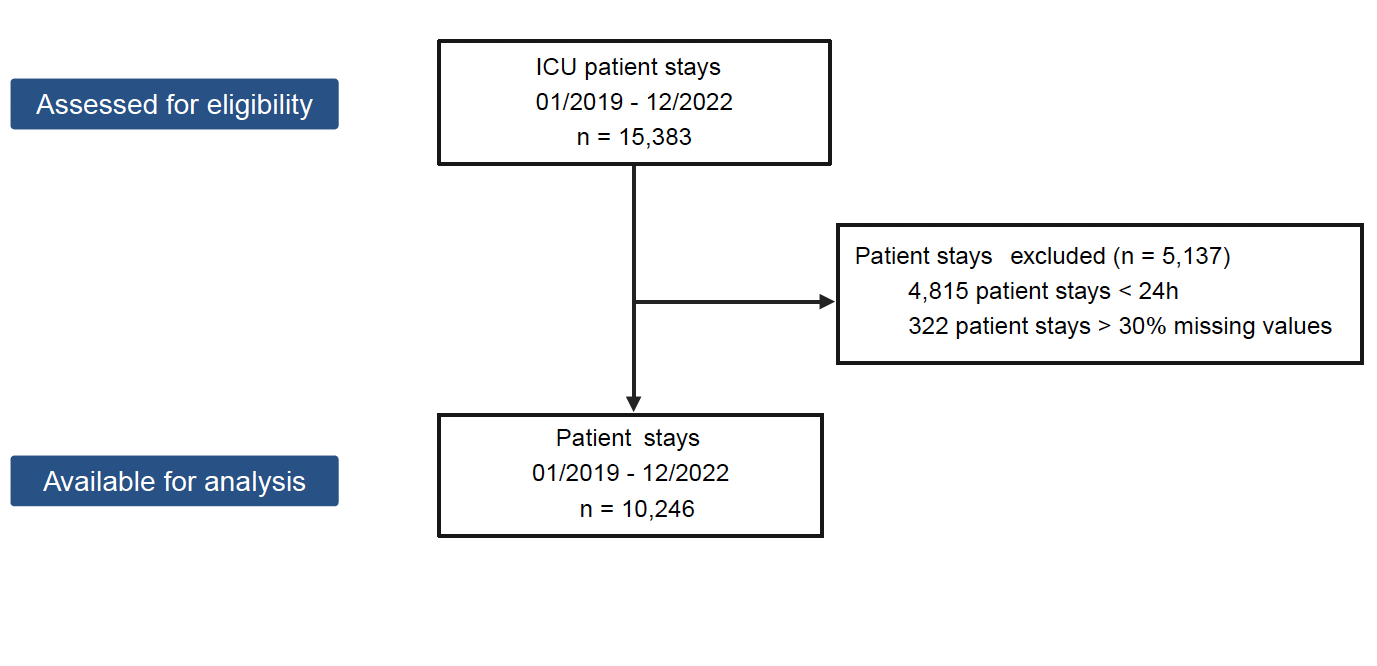


**b**, Flow chart for ICU stay days (defined as 24-hour intervals starting from ICU admission) included in the external validation.


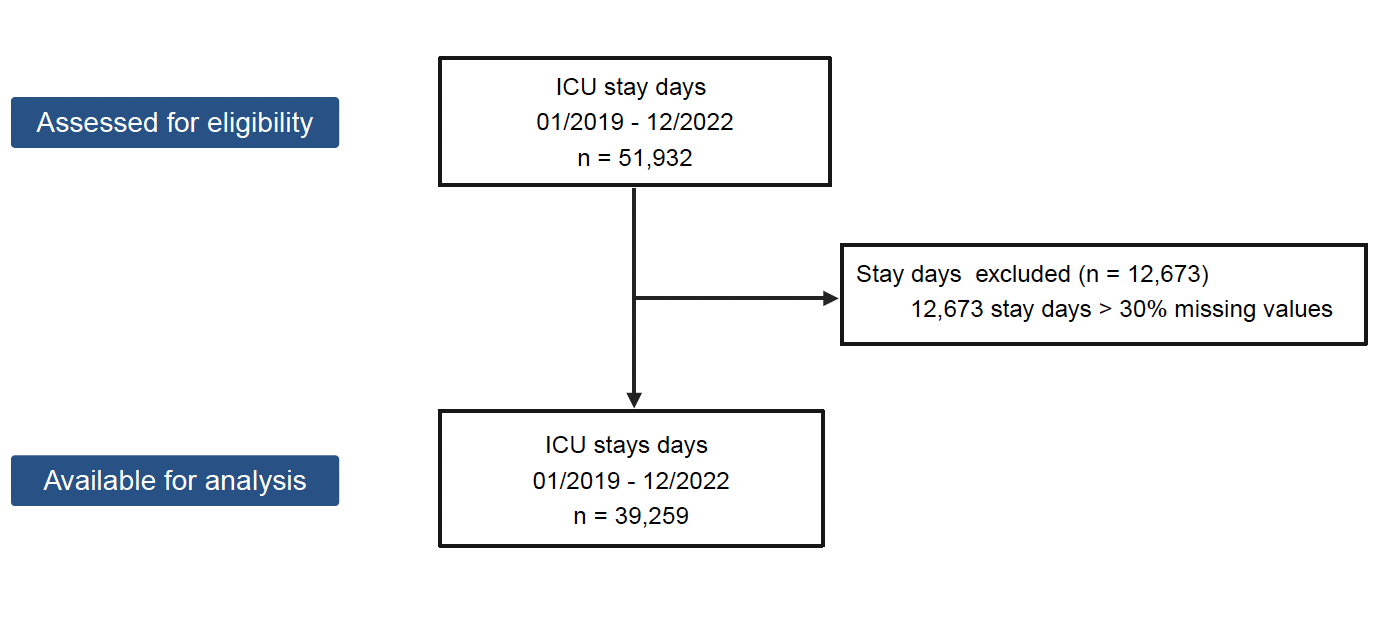


Supplemental Figure 5: *External validation of the LGBM-48h prediction model using the MIMIC-IV dataset.*


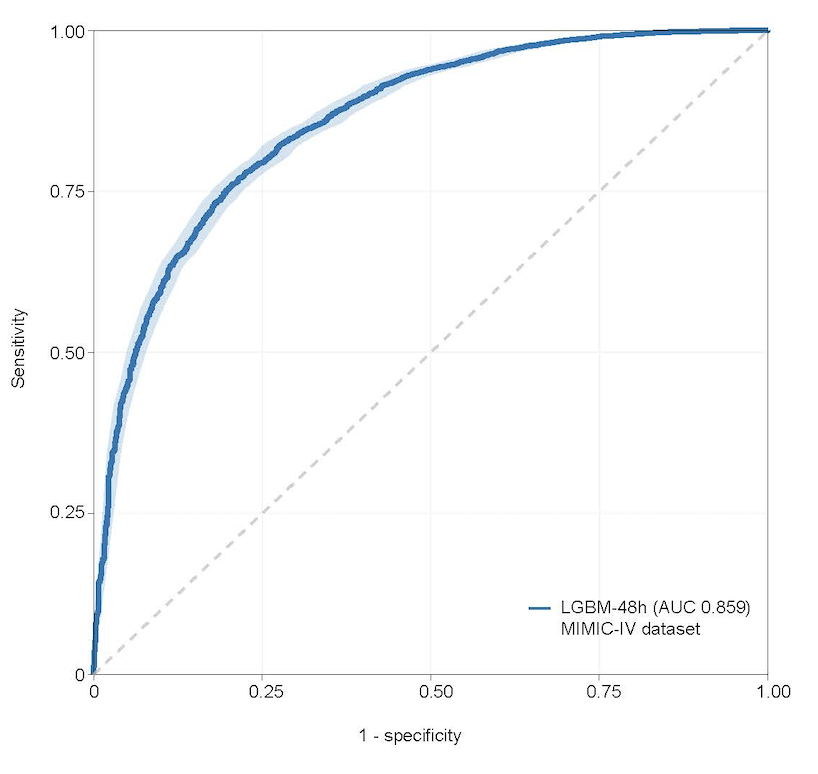


**Supplemental Figure 4:** ROC curve illustrates model performance with the original LGBM-48h algorithm applied to the MIMIC-IV dataset without retraining.

Supplemental Figure 6: *Cutoff selection for 48-hour mortality risk across the ICU stay in the MIMIC-IV dataset.*


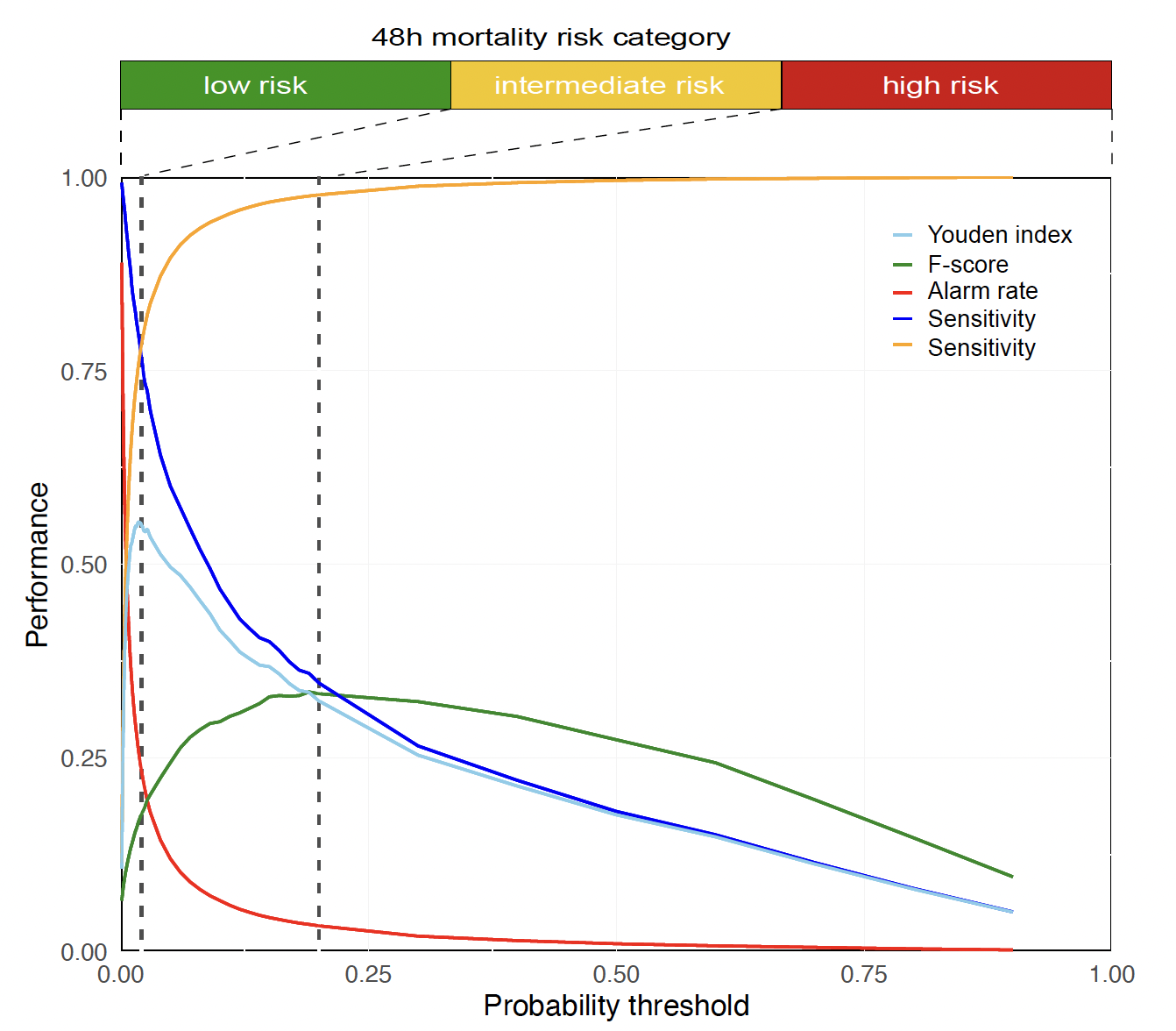


**Supplemental Figure 5:** Threshold plot illustrating Youden’s index, F-score, alarm rate, sensitivity, and specificity to define three risk categories for predicted 48-hour mortality in the **MIMIC-IV dataset**. The lower threshold was selected based on the maximum Youden’s index, while the upper threshold was selected based on the maximum F-score.
