# Supplementary material for: An interpretable machine learning algorithm enables dynamic 48-hour mortality prediction during an ICU stay
Source: Commun Med (Lond). 2025 Oct 15;5:426. doi: 10.1038/s43856-025-01192-z (PMC12528449; doi:10.1038/s43856-025-01192-z)
Supplement: Supplementary file 3 — Description of Additional Supplementary Data [file 43856_2025_1192_MOESM3_ESM.docx]

Description of additional supplementary file

File name: Supplementary Data

Description: Supplemental Data for Figures 1-5.
